# Comparing D-bar and common regularization-based methods for electrical impedance tomography

**DOI:** 10.1088/1361-6579/ab14aa

**Published:** 2019-04-26

**Authors:** S J Hamilton, W R B Lionheart, A Adler

**Affiliations:** 1 Department of Mathematics, Statistics, and Computer Science, Marquette University, Milwaukee, WI 53233, United States of America; 2 School of Mathematics, University of Manchester, Manchester, United Kingdom; 3 Department of Systems and Computer Engineering, Carleton University, Ottawa, ON, Canada

**Keywords:** electrical impedance tomography, GREIT, D-bar method, image reconstruction, conductivity

## Abstract

**Objective:**

To compare D-bar difference reconstruction with regularized linear reconstruction in electrical impedance tomography.

**Approach:**

A standard regularized linear approach using a Laplacian penalty and the GREIT method for comparison to the D-bar difference images. Simulated data was generated using a circular phantom with small objects, as well as a ‘Pac-Man’ shaped conductivity target. An L-curve method was used for parameter selection in both D-bar and the regularized methods.

**Main results:**

We found that the D-bar method had a more position independent point spread function, was less sensitive to errors in electrode position and behaved differently with respect to additive noise than the regularized methods.

**Significance:**

The results allow a novel pathway between traditional and D-bar algorithm comparison.

## 1. Introduction

Electrical impedance tomography (EIT) images the conductivity distribution within a body using body-surface measurements. Because electrical current propagates in a diffuse way, EIT is much less sensitive at depth than close to the electrodes. Reconstruction of EIT images is thus a challenging non-linear problem. Over the years, many EIT reconstruction methods have been proposed for 2D and 3D geometries, as well as difference, absolute, and frequency difference reconstructions. Two approaches to difference EIT reconstruction algorithms have been widely used in experimental studies in biomedical application (Adler *et al* 2012). One that gained wide popularity in the 1990s, Sheffield backprojection (Barber *et al* 1992), was implemented in the Sheffield and Goettingen EIT devices and reported in most of the early EIT experimental studies. Subsequently, reconstruction methods based on regularization techniques have become most widely used, and are distributed with EIT devices from Dräger, SenTec and Timpal. While in biomedical EIT difference imaging has been widely used mainly due to the difficulty in modeling body shape and electrode position, in geophysical applications of EIT difference data was typically not available and consequently absolute EIT reconstruction is common (Adler *et al* 2015). In this case, an accurate forward model is used and the absolute conductivity iteratively fitted to the data. Absolute EIT reconstruction was reported for the human chest (Newell *et al* 1992) but is still not widely used *in vivo*.

One relatively novel approach to 2D EIT image reconstruction is D-bar, a non-iterative absolute imaging approach (Nachman 1996, Isaacson *et al* 2004, Knudsen *et al* 2009). The literature on D-bar image reconstruction describes several potential advantages to other techniques, such as a robustness to errors in electrode positions and the body shape. The D-bar literature is rich, but there is little direct comparison of its performance to that of traditional (regularized) approaches.

The goal of our paper is thus to directly compare D-bar to other widely used EIT reconstruction algorithms. Since a general comparison is a vast problem, we have decided to limit this paper to consideration of the linearized difference EIT problem.

Comparison of algorithms is challenging, as there are multiple comparison criteria: resolution, position error, reconstruction shape accuracy, ability to suppress noise, ability to maintain sharp edges, resistance to electrode movement and other artefacts. In the following sections, we review the methods considered (section 2), discuss the comparison framework and criteria (section 3), present results (section 4), and analyze and discuss those results while drawing conclusions and suggesting further work in section 5.

## 2. Methods: reconstruction

We compare the results of three separate reconstruction methods: (1) the D-bar difference method, (2) generalized Tikhonov regularized linear difference imaging with a Laplacian penalty: or **RL** for regularized linear method, and (3) the GREIT method. Each method is briefly explained in this section. For notation, a difference EIT reconstruction calculates a vector of image elements, **x**, from a vector of difference EIT measurements, **y** = **v**_*σ*_ − **v**_ref_, between two frames of voltage measurements, **v**_*σ*_ and **v**_ref_.

### 2.1. The D-bar method for difference imaging

D-bar methods for EIT use nonlinear Fourier transforms specific to the EIT problem. The most common D-bar method (Nachman 1996, Isaacson *et al* 2004, Knudsen *et al* 2009) comes from transforming the conductivity equation

(1) ∇⋅σ∇u=0,

to a Schrödinger equation

(2) $$\left(-\nabla^{2}+q(z)\right)\;\tilde{u}(z)=0,$$

via the change of variables *ũ*(*z*) = *σ* l/2(*z*)*u*(*z*) where $q(z)=\nabla^{2}\sqrt{\sigma (z)}/\sqrt{\sigma (z)}$ for $z\in \Omega \subset {\mathbb{R}}^{2}$, and ∇^2^ denotes the Laplacian operator. This Schrödinger equation (2) can be solved using a D-bar method (Beals and Coifman 1985) which introduces an auxiliary parameter k∈ℂ and uses special solutions *ψ*(*z, k*) to

(3) $$\left(-\nabla^{2}+q(z)\right)\;\psi (z,k)=0,$$

asymptotic to *e*^*ikz*^ for large |*k*| or |*z*|. We associate ${\mathbb{R}}^{2}$ with ℂ via *z* = (*z*_1_, *z*_2_) ↦ *z*_1_ + *iz*_2_ here so *kz* is the complex product. The solution process involves using a special transform, which can be thought of as a nonlinear Fourier transform, specific to this problem (3). The breakthrough for EIT is that this special nonlinear Fourier data (called Scattering data), can be computed from current and voltage measurement data. Then, the conductivity can be recovered using the inverse transform.

Difference imaging with the D-bar method uses a modified scattering transform, called the *differencing scattering transform* (Isaacson *et al* 2006). The process is

$$\underset{\left(\Lambda_{\sigma },\Lambda_{\sigma_{\text{ref}}}\right)}{\text{Current/Voltage Data}}\;\overset{1}{\to }\text{ , }\underset{t_{R}^{\text{diff}}(k)}{\text{Scattering Data}}\;\overset{2}{\to }\;\underset{\sigma^{\text{diff}}\left(z\right)}{\text{Conductivity}}.$$

**Step 1:** Compute the low-pass differencing scattering data $t_{R}^{\text{diff}}(k)$. For each k∈ℂ\{0}, evaluate the approximate scattering data

(4) $$t_{R}^{\text{diff}}(k)=\{\begin{array}{ll} \frac{1}{\sigma_{b}}\int_{\partial \Omega }e^{i\bar{k}\bar{z}}\left(\Lambda_{\sigma }-\Lambda_{\text{ref}}\right)e^{ikz}dS(z), & 0<\left|k\right|⩽R\\ 0 & \left|k\right|>R \end{array}$$

where *σ*_*b*_ denotes the best constant conductivity approximation to the conductivity near the boundary, Λ_*σ*_ and Λ_ref_ are the Dirichlet-to-Neumann (DN) maps corresponding to the two frames of voltage measurements **v**_*σ*_ and **v**_ref_, respectively, for the chosen applied current patterns. Matrix approximations to the DN maps can be formed using discrete inner products (see Isaacson *et al* (2004).

**Step 2:** Recover the low-pass conductivity *σ*^diff^ (*z*). For each *z* ∈ Ω, solve the D-bar equation via the integral equation

(5) $$\mu_{R}^{\text{diff}}(z,k)=1+\frac{1}{4\pi^{2}}\underset{\mathbb{C}}{\int }\frac{t_{R}^{\text{diff}}\left(k^{\prime }\right)e^{-i\left(k^{\prime }z+\bar{k^{\prime }}\bar{z}\right)}}{\left(k-k^{\prime }\right)\bar{k^{\prime }}}\bar{\mu_{R}^{\text{diff}}\left(z,k^{\prime }\right)}dk_{1}^{\prime }dk_{2}^{\prime },$$

and recover the low-pass D-bar difference conductivity

(6) $$\sigma^{\text{diff}}(z)=\sigma_{b}{\left[\mu_{R}^{\text{diff}}(z,0)\right]}^{2}-\sigma_{b}$$

which corresponds to the reconstructed image, **x**, in other methods.

The parameter *R* is considered the regularization parameter in the D-bar method as it controls the radius of the low-pass filter in the nonlinear Fourier domain. For additional stability, a thresholding is also commonly used by setting $t_{R}^{\text{diff}}\left(k\right)=0$ if either $\left|\text{Re}\left\{t_{R}^{\text{diff}}(k)\right\}\right|$ *or $\left|\text{Im}\left\{t_{R}^{\text{diff}}(k)\right\}\right|$* is greater than a chosen threshold. The thresholding helps to control blowup in the scattering domain where neighboring pixels can differ by a factor of 10, 100, etc. Note that Step 2 is the inverse transform step, whereas in Step 1 we bypass the full plane ${\mathbb{R}}^{2}$ formulation of the forward transform by instead computing the scattering data from a boundary integral equivalent through integration by parts.

#### 2.1.1. Computational notes

In practice, equations (4) and (5) are discretized and computed with matrices. Note that the integral equation in (5) can be written using convolutions:

(7) $$\mu_{R}^{\text{diff}}(z,k)=1+\frac{1}{\pi k}*\left(\frac{t_{R}^{\text{diff}}(k)e^{-i(kz+\bar{k}\bar{z})}\bar{\mu_{R}^{\text{diff}}(z,k)}}{4\pi \bar{k}}\right),$$

where * denotes convolution over k∈ℂ. Therefore, we can solve the integral equation (5) using fast Fourier transforms (FFTs) as in Vainikko (2000), Knudsen *et al* (2004) and Mueller and Siltanen (2012). We use a uniformly spaced *k*-grid on a square [− *D*_*k*_*, D*_*k*_)^2^, where *D*_*k*_ ⩾ *R*, of size *M* × *M*, where *M* is a power of 2, and the grid-size is *h*_*k*_ = 2*D*_*k*_*/*(*M* − 1). This *k*-grid defines the points where we compute the scattering data$t_{R}^{\text{diff}}\left(k\right)$. For the reconstructed image *σ*^diff^ (*z*), the computational *z*-grid is very flexible since the solution to the D-bar equation is computed point-wise. One can use whatever type of grid is most appropriate for the task: uniformly spaced, non-uniformly spaced, FEM mesh, etc.

The evaluation of the scattering transform $t_{R}^{\text{diff}}\left(k\right)$ in Step 1 requires knowledge of how the DN maps Λ_*σ*_ and Λ_ref_ act on the exponential function *e*^*ikz*^ for *z* ∈ ∂Ω. We approximate this by using the discrete matrix approximations **L**_*σ*_ = (**R**_*σ*_) ^−1^ and **L**_ref_ = (**R**_ref_) ^−1^, where, e.g.

(8) $$R_{\sigma }(m,n):=\overset{L}{\underset{l=1}{\sum }}\frac{\phi_{l}^{m}v_{l}^{n}}{\left|e_{l}\right|},\;1⩽m,n,⩽{\text{num}}_{LI},\;1⩽l⩽L,$$

where {*ϕ*^*m*^} and {*v*^*n*^} are the normalized current, and voltage, patterns respectively, num_*LI*_ denotes the number of linearly independent current patterns applied, *L* the number of electrodes used, and |*e*_*ℓ*_| denotes the area of the *ℓ* th electrode. We then expand the asymptotic behavior *e*^*ikz*^, at the centers of the electrodes *z*_*ℓ*_, in the orthonormal basis of normalized current patterns {*ϕ*^*m*^} as

(9) $$e^{ikz_{l}}\approx \overset{{\text{num}}_{LI}}{\underset{m=1}{\sum }}a_{m}(k)\phi_{l}^{m}.$$

Discretizing (4) using a simple Simpson’s type rule gives

(10) $$t_{R}^{\text{diff}}(k)\approx \{\begin{array}{ll} \frac{1}{\sigma_{b}}\frac{P}{L}e^{i\bar{k}\bar{z}}\Phi \left(L_{\sigma }-L_{\text{ref}}\right)a(k), & 0<\left|k\right|⩽R\\ 0 & \left|k\right|>R, \end{array}$$

where *P* is the perimeter of the domain Ω, $z\in {\mathbb{C}}^{1\times L}$ is the row vector of positions of the centers of the electrodes, Φ the orthonormal matrix of normalized current patterns *ϕ*^*m*^, and **a**(*k*) the vector of coefficients in the expansion (9).

To solve the D-bar equation, and recover the D-bar conductivity *σ*^diff^, the integral equation must be solved for each *z* point in your chosen mesh. Using convolution, (5) can be written as (7), and thus can be written as a linear system

(11) $$[I-AT(\bar{\cdot })]\mu^{\text{diff}}=1,$$

for each value of *z*, where A and T are defined by their actions via

$$Ag(k)=\frac{1}{\pi k}*g(k),\text{ and }Tf(k)=\frac{t_{R}^{\text{diff}}(k)e^{-i(kz+\bar{k}\bar{z})}}{4\pi \bar{k}}f(k).$$

The convolutions can be computed using 2D fast Fourier transforms as

$$\frac{1}{\pi k}*g(k)=h_{k}^{2}\text{ IFFT2 }\left[\text{FFT}2\;\left(\frac{1}{\pi k}\right)\text{ FFT}2(f(k))\right],$$

and thus the linear system (11) solved using a matrix-free solver such as gmres, separating the real and imaginary parts. For further details of the numerical implementation of the D-bar method the interested reader is referred to Mueller and Siltanen (2012) and Hamilton *et al* (2018).

### 2.2. RL

Tikhonov regularization-based approaches to EIT were developed in the 1980s, e.g. Yorkey (1986). The key idea is to separate the reconstruction into a ‘forward’ and an ‘inverse’ problem. First, the body region is discretized into elements that map to a finite element grid, and represented as a vector, ***σ***.

Linear difference EIT uses as data a change, **Δ*σ*** = ***σ*** – ***σ***_ref_, between a time of interest, ***σ***, and a reference instant ***σ***_ref_, which we model as homogeneous.

A frame of voltage measurement data, **v**, is acquired through a set of drive and measurement patterns. Measurement data are simulated using a forward problem, *F*(·), typically using a FEM: **v**_*σ*_ = *F*(***σ***) and **v**_ref_ = *F*(***σ***_ref_*)*, from which the measurement change vector, **y** = **v**_*σ*_ − **v**_ref_ is calculated.

Differences from the reference value of the discrete conductivity in the forward model ***σ*** are parametrized by a coarse-to-fine map, **Δ*σ*** = **Mx**, where **x** is the vector of image voxel values. Here, each element, **M**_*ij*_, represents the volume fraction of forward model element *i* contained within the image element *j*. Since the forward model requires a high density of mesh parameterization in areas near the electrodes (Grychtol and Adler 2013). Using the map **M**, we parameterize the body onto the reconstruction mesh.

The sensitivity of measurement *i* to changes in voxel element *j*, is then given by the matrix, **J**_*ij*_ = *∂***y**_*i*_/∂**x**_*j*_ evaluated at ***σ***_ref_.

As **J** is a severely ill conditioned matrix (Breckon and Pidcock 1988), rather than simply solving for **x**, reconstruction methods seek an **x** to minimize

(12) $$\|Jx-y{\|}^{2}+\lambda \Psi^{2}(x),$$

where Ψ is a regularizing penalty term, and the regularization hyperparameter λ > 0 controls the trade-off between fitting the data of the linearized problem and satisfying the *a priori* assumption that Ψ (*x*) is small. To enforce a smoothing assumption on the images we choose Ψ (*x*) = ||*L***x** ||_2_ where *L***x** is an approximation to the Laplacian of the conductivity. This corresponds also in the Bayesian formulation to the MAP estimate when the errors in the data are assumed to be Gaussian and uncorrelated with equal variance, and the prior distribution is a generalized multivariate Gaussian with inverse covariance matrix proportional to *L*^*T*^*L*. The generalized Tikhonov regularized solution to the Regularized Linear(RL) problem is given by

(13) $$x_{\text{LR}}={\left(J^{T}J+\lambda L^{T}L\right)}^{-1}J^{T}y.$$

Other common choices for regularization penalty terms in EIT include truncated singular value decomposition, and Total Variation. For further details see Adler *et al* (2015), and the references therein, as well as other chapters in the same work.

#### 2.2.1. GREIT

The GREIT algorithm (Adler *et al* 2009) is a type of regularized image reconstruction in which the values of the reconstruction parameters are set in a systematic way, from a set of desired characteristics defined by the authors.

We use the formulation of GREIT developed by Grychtol *et al* (2016), which we briefly review to illustrate the relevant choices. Linear algorithms for difference EIT represent image reconstruction by a reconstruction matrix, **R**, which calculates a reconstructed image **x** = **Ry**, from difference data, **y**. The GREIT reconstruction matrix minimizes an error *ϵ*^2^(**R**) = E [‖**x** − **Ry**‖^2^]. The expectation, E[·] is over a distribution of ‘training’ targets, **t** ^(*i*)^, for which the corresponding data, **y**^(*i*)^, and a ‘desired’ image, **x**^(*i*)^ = **Dt**^(*i*)^, are calculated, where **D** is the ‘desired image’ matrix, which maps each training sample location onto a larger image region. The reconstruction matrix which minimizes *ϵ* is **R** = E [**xy**^*T*^] (E [**yy**^*T*^]) ^−1^.

Given a distribution $t\sim N\left(0,\Sigma_{t}\right)$ of training targets and noise $n\sim N\left(0,\Sigma_{n}\right)$,

(14) $$R=D\Sigma_{t}^{-1}J^{T}{\left(J\Sigma_{t}^{-1}J^{T}+\lambda \Sigma_{n}\right)}^{-1}.$$

The parameter λ is selected so that noise performance of the reconstruction matrix matches a selected ‘noise figure’ (NF) value.

## 3. Methods: evaluation

Here we present the simulated phantoms used for the experiments, as well as figures of merit that will be used to evaluate and compare the various reconstruction methods. Since we plan to compare D-bar to linear difference reconstructions, we choose phantoms with very small contrasts (**Δ***σ*/*σ* ⩽ 0.1) for which the linearized problem is a good approximation.

### 3.1. Simulation models

**Figure 1.**
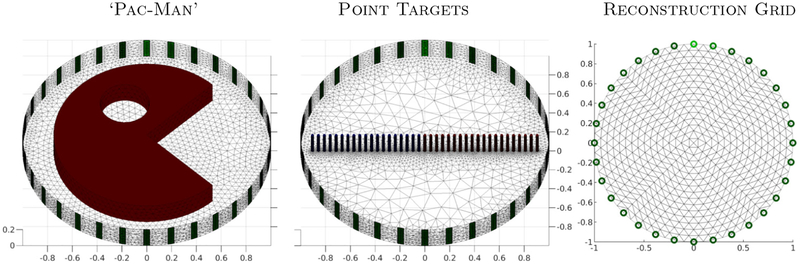
Phantoms: ‘Pac-Man’ shape (left), point targets (middle), and reconstruction grid. Two scenarios are considered for the point targets phantom. The first tracks the response of each reconstruction method to a single point target as it moves across the domain. The second explores the algorithms’ responses two two point targets located close together versus further away.

We examined the behavior of the algorithms on three different phantoms: ‘Pac-Man’, a small single point target, and two point targets (see figure 1). These three targets have quite different characteristics; ‘Pac-Man’ has sharp edges and a hole, the single point target example studies a point target moving from the center of the domain to the outside, and the two point targets start close to each other in the center of the domain and move away from each other towards the boundary. Small contrasts were used in this study, 0.1 × the background value. ‘Skip-4’ stimulation was simulated, using 32 equally spaced electrodes of width 0.05 m, with monopolar voltage measurements on all electrodes (including the driven electrodes). All algorithms computed difference image reconstructions on the FEM reconstruction grid shown in figure 1 (right).

In order to reduce the possibility of an ‘inverse crime’ simulation and reconstruction models were intentionally different. Simulation models were three dimensional, based on a complete electrode model, and used finite element models based on 9800 (‘Pac-Man’) and 94 400 (‘moving targets’) vertices. All 3D models were circular with a radius of 1 m and a height of 0.2 m, and with a background conductivity of 1.0 S m −1. The ‘Pac-Man’ region had a radius of 0.75 with a 90° ‘mouth’ and an ‘eye’ of radius 0.2 m, centered half-way (0.375) between the center and the region edge. The point targets were cylinders of radius 0.01 m a height 0.2 m spaced by $\frac{1}{21}$ of the region radius on each side of the center. The reconstruction mesh was a regular 2D mesh with 1024 elements and 545 vertices and used point electrodes.

### 3.2. Figures of merit

Most EIT reconstruction methods allow control of the trade-off between resolution and noise performance. We use the term ‘hyperparameter’ for the parameter which controls this behavior. For, D-bar, the parameter is the radius *R* of the admissible scattering data in (4). Regularized techniques use a hyperparameter to control the weighting of the regularizing penalty function. In RL, this hyperparameter is λ, while for GREIT this hyperparameters is typically converted into a noise figure (NF) value.

Since each method has an independent parameter space, a ‘fair’ method to select comparable values was needed. We chose a method based on the ‘L curve’ (Hansen and O’ Leary 1993). We use the notation that a reconstruction method at hyperparameter value λ calculates an image **x**_λ_ from difference EIT data **y**. We then find the best fitting multiplicative factor *f*_λ_ which minimizes the norm

(15) $$D_{\lambda }(x)=\|F\left(\sigma_{\text{ref }}+f_{\lambda }x_{\lambda }\right)-F\left(\sigma_{\text{ref }}\right)-y{\|}_{2}.$$

For each reconstructed image we calculated two norms, an image norm Ψ_λ_ = ‖*L***x**‖_2_ (equal to the regularization penalty function), and a data misfit *D*_λ_. We chose *L* as a matrix formulation of the discrete Laplacian on the reconstruction FEM and the ‖ · ‖_2_ norm. We note that these norms are the ones used in the RL algorithm, which thus had an ‘advantage’ in the sense that it was formulated to minimize the norms against which it is subsequently evaluated.

Next, we plotted Ψ_λ_ against *D*_λ_ and selected λ_*m*_ as the hyperparameter value at the L-curve corner. Since in EIT the L-curve minimum is typically over-regularized with respect to a visual selection, we also chose values, λ_2*m*_, λ_3*m*_, and λ_4*m*_, where λ_*Km*_ was chosen so that the image norm $\Psi_{\lambda_{Km}}=K\Psi_{\lambda_{m}}$ was a multiple of the L-curve minimum. Using the zero noise ‘Pac-Man’ data, the parameter values λ_m_, λ_2*m*_*, λ*_3*m*_, and λ_4*m*_ were chosen and then held fixed across all other experiments. The parameters were thus fixed at λ = 46.4 × 10^−3^, λ = 5.41 × 10^−3^, λ = 0.903 × 10^−3^, and λ = 0.215 × 10^−3^ for the RL method, NF = 0.921, NF = 3.43, NF = 9.19, and NF = 36.4 for GREIT, and *R* = 4.0, *R* = 5.6, *R* = 6.6, and *R* = 7.6 for the D-bar method.

**Figure 2.**
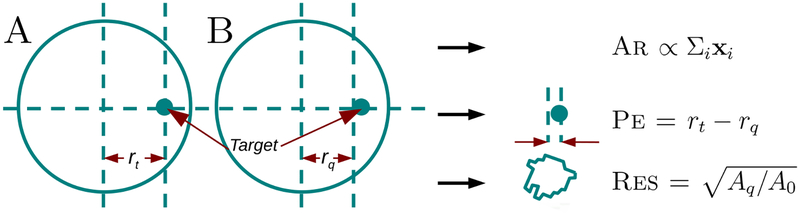
Illustration of figures of merit used. A: Reconstructed image with position of the simulated target, *r*_*t*_. B: Thresholded reconstructed image, with center of gravity, *r*_*q*_.

In order to evaluate reconstruction algorithm performance, various figures of merit (FoM) have been proposed over the years. We chose FoM which were proposed in Adler *et al* (2009) and have subsequently seen fairly wide application (figure 2). For this calculation, small targets were simulated at known radial positions, *r*_*t*_ in a cylindrical medium. From each reconstructed image, A, a threshold was chosen at $\frac{1}{4}$ of the maximum difference, and a thresholded-image, B calculated. The center of gravity of B is *r*_*q*_ and its area *A*_*q*_. We used the following parameters: Ar (amplitude response) equal to the sum of all image elements (scaled so the center target is 1), Pe (position error) the difference in original to reconstructed position, and Re s (resolution) the square root of the resolution ratio compared to the medium (*π*).

## 4. Results

**Figure 3.**
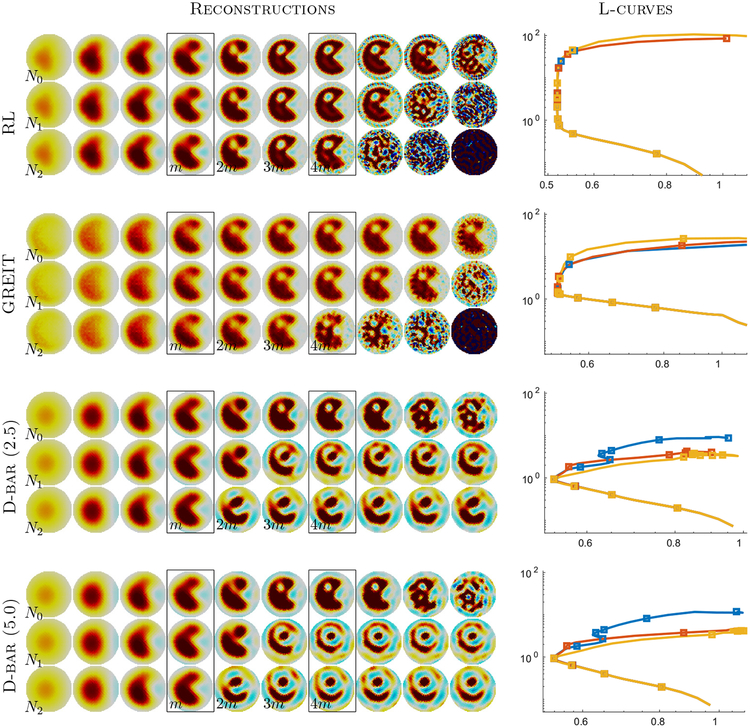
*Left:* Reconstructed difference images displayed for varying hyperparameters from smoothest reconstruction to least smoothed for the RL, GREIT, and D-bar methods. D-bar reconstructions are shown for a fixed threshold of 2.5 as well as 5.0. Each row in the respective subfigures corresponds to a different data noise level. The boxed images (*m*) correspond to the L-curve minimum selected from the corresponding L-curve shown on the right. Images 2*m*, 3*m*, and 4*m* correspond to reconstructions whose image norms are 2, 3, and 4 times the L-curve minimum. *Right:* The ‘L curve’ of data-norm (horizontal axis) versus the image-norm (vertical axis) with each square marker corresponding to a reconstruction on the left for the noise levels: *N*_0_: No noise (yellow), *N*_1_: SNR = 10^5^ (blue), and *N*^2^: SNR = 10^4^ (red).

The first step was an analysis of reconstructions parameter values using the L-curve approach, as shown in figure 3. For each reconstruction method (and two variants of the D-bar method) images were calculated across a large range of hyperparameter values. Data were simulated using the ‘Pac-Man’ model (1) with no-noise (*N*_0_) and two levels of added noise (*N*_1_; *N*_2_). To ensure comparability, the same noise values were used for all images. For each reconstruction method, ten representative hyperparameter values were chosen corresponding to the L-curve minimum, *m*, and its multiples, as well as examples of extremely smooth (left images) and noisy (right images).

We note that the L-curve shape displays a ‘folded’ pattern in which the noisiest images have an increased data fit in comparison to the L-curve minimum. This effect is explained by the mismatch between the forward and reconstruction models, and is most severe for D-bar, which does not perform an explicit fitting of a forward model.

The visual patterns are reflective of the details of each method. For *N*_0_, the pattern of noise at the right reflects the effect of model mismatch. This effect is seen as a boundary artefact for RL and as a more interior noise in GREIT. The pattern of noise is also central, and has a lower spatial frequency in D-bar, and this depends on the threshold chosen. Thresholds of 2.5 and 5.0 were used. The accuracy with which the methods were able to reconstruct features of the target varies across algorithms. For example, the edges of the ‘Pac-Man’ ‘mouth’ were best reconstructed by RL, and this effect was likely due to the closeness of match of the RL forward and inverse models. For the noisy images, *N*_1_ and *N*_2_, the visual effect became more severe for as the hyperparameter increased from *m* up to 4m. Again the visual pattern of the projected noise had a different behavior in D-bar versus the regularized algorithms.

**Figure 4.**
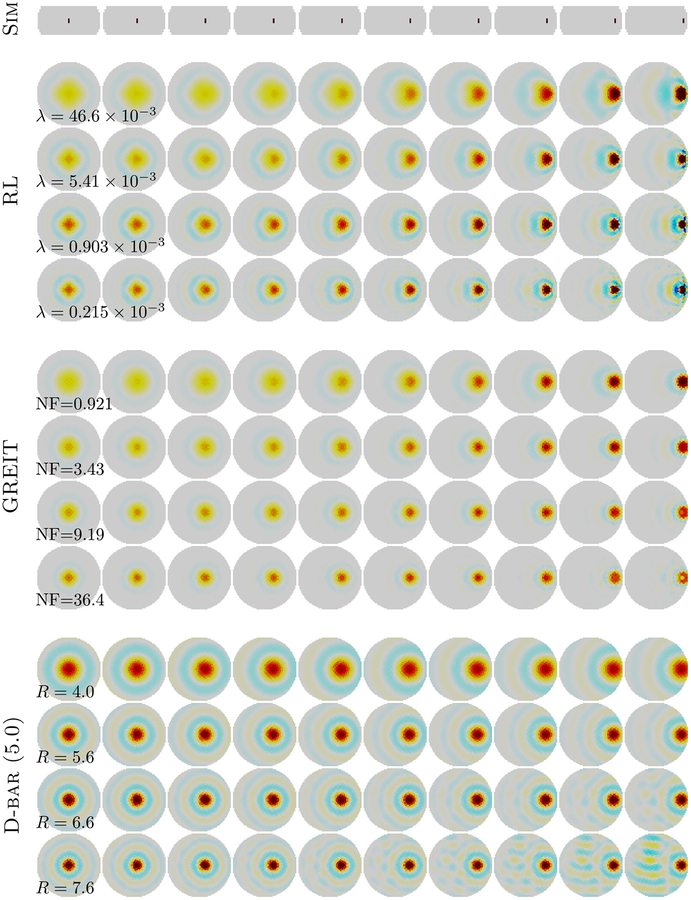
Images as a function of position using the single moving target phantom in figure 1. Simulated target positions are shown above (Sim). Reconstructions are compared for RL (first), GREIT (second), and D-bar with a threshold of 5.0 (third). Fixed parameter values of λ, NF, and *R* were used for the algorithms.

To explore the spatial variation in image reconstruction performance, figure 4 shows the images reconstructed for small targets moving from the center to the edge of the domain. For all methods, as expected, the resolution is relatively low at *m*, but improves as the image norm is allowed to increase 2*m* … 4*m*. We note that D-bar shows a very spatially uniform reconstruction: both the resolution and the ‘ringing’ region around it is extremely uniform with position. The RL method shows a characteristic improvement in resolution toward the boundary, and also displays a changing spatial pattern with a increase in the level of ringing with a smaller reconstructed target near the boundary. The GREIT algorithm shows somewhat more uniform spatial resolution than RL, but less so than D-bar. It also shows much lower levels of ringing, as is expected since this was a key design requirement for the algorithm.

**Figure 5.**
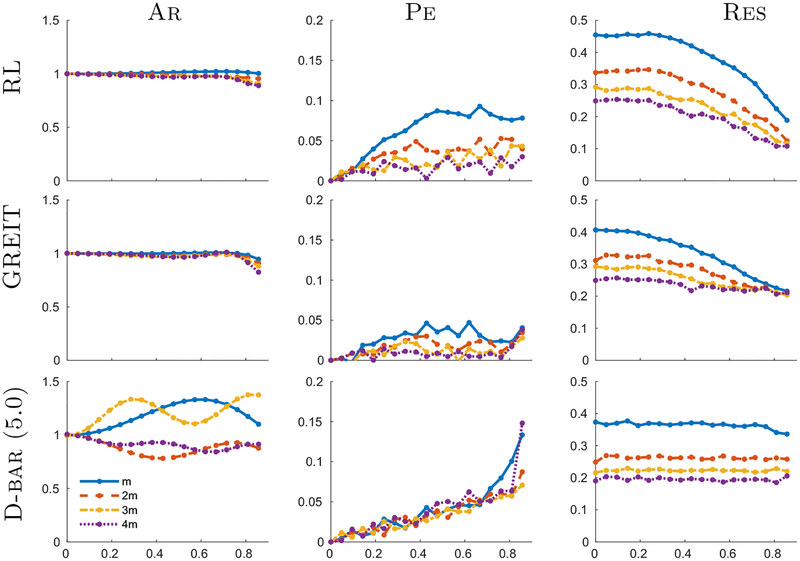
Figures of merit for the reconstructions of the single point-targets shown in figure 4, computed as a function of radial position (horizontal axis with center 0 and boundary 1).

To quantify the image reconstruction characteristics of figure 4, we calculated figures of merit for the amplitude (Ar), position error (Pe) and resolution (Res) (figure 5). Ar is roughly uniform for regularized algorithms, but is less uniform at the hyperparameters corresponding to 4*m* than *m*. Here D-bar has an oscillating Ar behavior with a spatial frequency that increased with image resolution. This spatially-varying behavior appears to be due to the ringing in the D-bar images; as a part of the otherwise spatially-uniform image response is ‘cut’ outside the domain, the Ar varies with the amplitude of the ringing.

Pe was fairly low for all methods and increased toward the medium boundary. Pe was lower for GREIT than RL, again because this is a design requirement for the method. For both regularized methods, Pe was higher for hyperparameters corresponding to *m* than 4*m*, largely due to the increased Res. On the other hand, D-bar showed a very uniform Pe with both radial position and with hyperparameter level, except for right next to the boundary for some hyperparameter values. Using a calibration factor (i.e. spatially scaling the image by about 5%), it would be possible to create D-bar images with very low Pe. The Res for the regularized methods was large in the center but decreased (improved resolution) toward the boundary. This effect was less visible in GREIT than RL, because GREIT explicitly seeks to achieve uniform, rather than small, Res. This spatially-varying behavior was not seen in D-bar, which had extremely uniform resolution at all radial positions.

**Figure 6.**
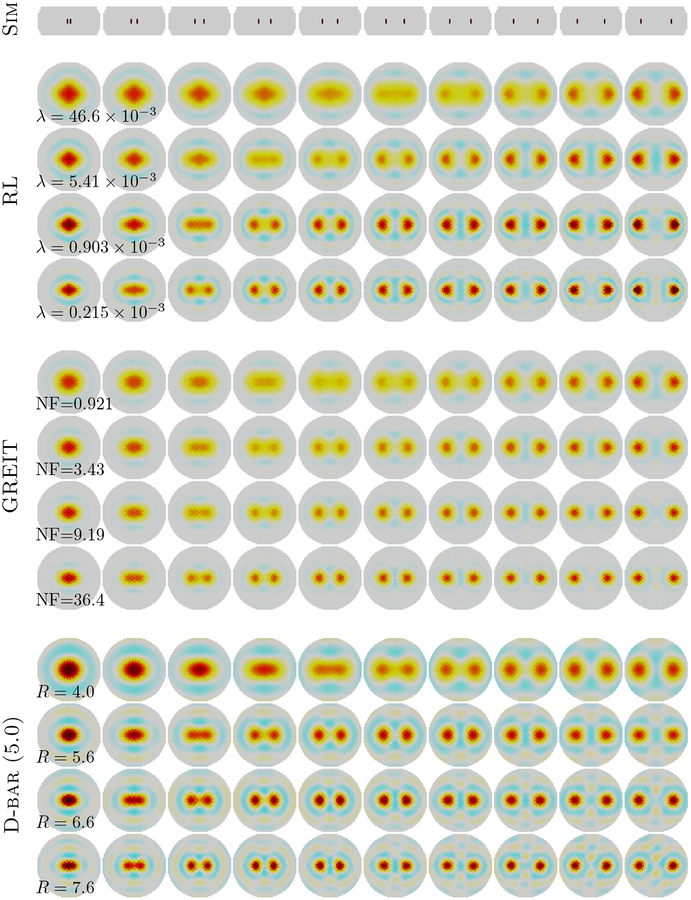
Comparison of reconstructions with a two moving targets for RL (first), GREIT (second), and D-bar with a threshold of 5.0 (third) with fixed parameter (λ, NF, *R*) values corresponding. Simulated target positions are shown above (Sim).

The ability of EIT to resolve separate objects was determined by the resolution and also influenced by image reconstruction features such as ringing. Figure 6 shows the images as a function of target separation. Using the point target phantom, figure 1, targets were simulated at opposite radial positions, moving away from each other. The resulting images show the resolving ability of each algorithm as a function of hyperparameter. There is a clear influence of both the point resolution and the ringing in each case.

**Figure 7.**
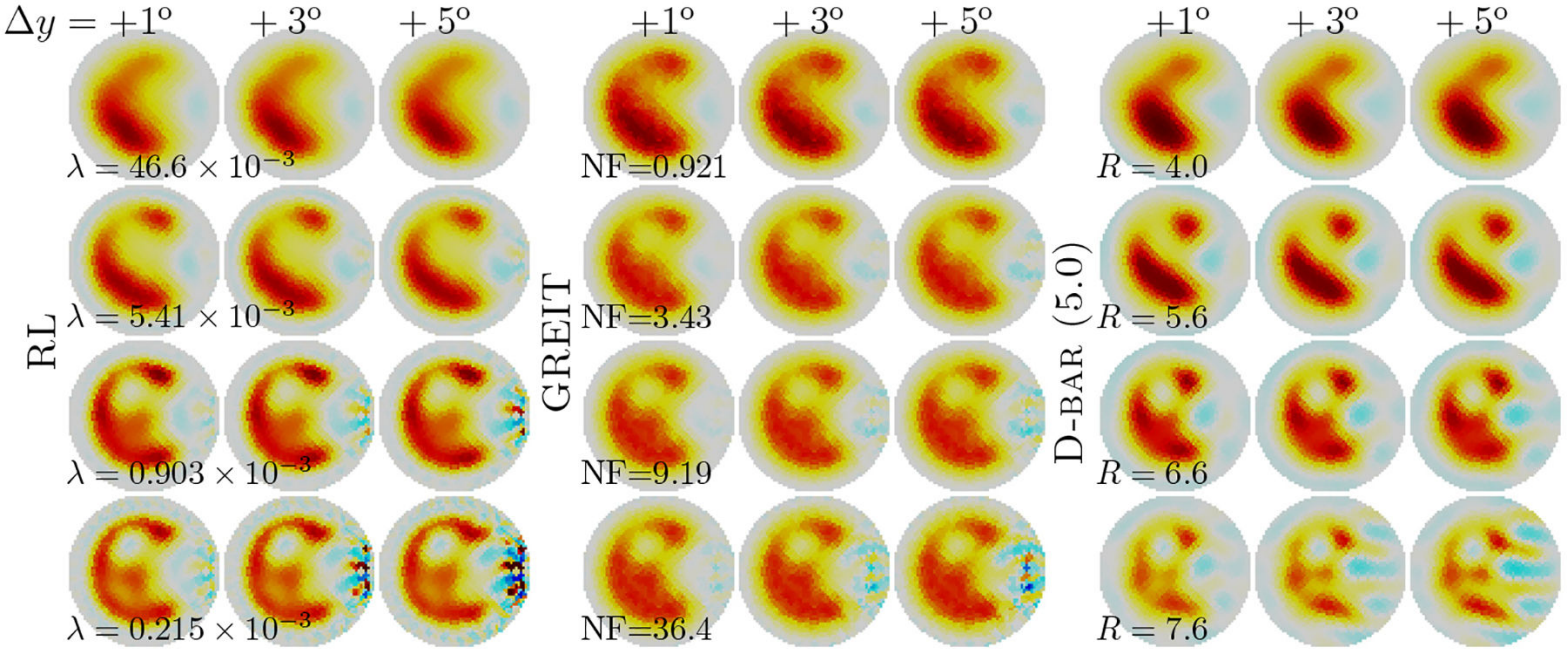
Reconstructions of an electrode position error for the ‘Pac-Man’ phantom. The electrode at the ‘mouth’ was moved between voltage measurements by the indicated amount (degrees). Fixed values of the regularization parameters (λ, NF, *R*) were used.

Lastly, we explored the ability to reconstruct difference images where electrodes move between measurements, as shown in figure 7. These figures reconstructed data from the ‘Pac-Man’ phantom, in which the electrode in the center of the ‘mouth’ was moved between the *V*_*σ*_ and *V*_ref_ measurements. These reconstructions evaluated the ability of the algorithms to manage data with uncertainty in the electrode positions for four fixed regularization parameters. For all methods, with the hyperparameter corresponding to *m*, very little effect of electrode movement was seen, but the effect increased and was visible for all methods at 4*m*. Overall, the influence on the reconstructed image is greatest for the RL algorithm and least for D-bar when looking at the response of the algorithms to only electrode displacement. For the regularized approaches, the electrode movement effect was seen largely at the medium boundary, while for D-bar there effect appeared to move inside the domain as well (only for 4*m*).

## 5. Discussion and conclusions

In this paper we compared three reconstruction methods for 2D difference EIT that are linear once regularization parameters are chosen. While GREIT and RL explicitly trade off data fit of the linearized problem against a penalty on the image term, D-bar uses an explicit theoretically devised approximate inverse where regularization is applied at an intermediate step.

Our analysis in this paper was limited to the region where linear difference EIT reconstruction is valid. All simulated contrasts were constrained to be small to ensure this validity. This means that this paper does not explore the very interesting comparison of D-bar and iterative regularized methods in cases where the non-linearities are important. The small level of contrasts also explains why very small levels of noise (SNR = 10^5^) have a perceptible influence on the reconstructed images.

Numerous differences were seen between the reconstruction behavior of D-bar and that of regularized algorithms. To our knowledge, we are the first to observe these effects and thus cannot validate them against other reports. In many cases, the behavior is consistent with our understanding of the mathematics of the methods; however, in some cases these differences are less well understood and would merit further study, see end of this section.

The GREIT and RL methods have a position dependent resolution operator. Since the sensitivity of boundary measurements to a conductivity change decreases with distance from the driven and measurement electrodes, these methods compensate for the lack of information in the measurements by applying the *a priori* information included in the regularization term which results in broader point spread function. The effect of noise in the data on the reconstructed images is very different in the case of D-bar and regularized approaches. RL (and to a lesser extent) GREIT ‘project’ noise to the image boundary, while the noise in the D-bar images is roughly uniformly distributed. In regularized algorithms, this boundary effect can be explained by the increased sensitivity of EIT near the electrodes; if a method wants to ‘explain’ measurement noise, it can do it most economically using contrasts at the boundary.

Measurements near the boundary are inevitably much more sensitive to changes in electrode position and changes in the boundary shape than they are to conductivity changes deep in the body. In Lionheart (1999) the case is made that the dimension has the biggest effect and 2D data will generally not fit a 2D model. It is also claimed that one needs to get the shape and electrode positions correct before one can expect to use the measurements to fit the conductivity. However that is not the whole story as the boundary voltage data, in the linear approximation, contains some components that are related only to conductivity changes and not confounded by shape and electrode position error (Lionheart 1999, Boyle *et al* 2012). We observed that D-bar appears to be much less sensitive to electrode position errors than regularized reconstructions, holding the regularization parameter fixed. Future work will explore and quantify the effect of boundary shape errors across methods, in particular for the D-bar method.

In this work, we present numerical evidence for these properties of D-bar, but we hope that a greater theoretical understanding will follow in the future. If we can understand theoretically the approximate position invariant point spread function and the robustness to electrode position error in D-bar difference imaging, then there is a hope that it will spur the development of 3D EIT reconstruction methods with the same qualities.

D-bar methods use a complete set of voltage data from the system of electrodes, and approximate the continuum Dirichlet-to-Neumann map from those measurements. In this paper, we used this complete data set for all methods, whereas several biomedical EIT systems discard the voltages on driven electrodes. Future research will include studying the effect of interpolating this missing data using *a priori* assumptions about the conductivity near the boundary.

Regularized linear difference methods, as well as the regularized non-linear fitting methods, are derived from systematic assumptions about the noise distribution in the data and *a priori* assumptions about the image. By contrast D-bar methods use an explicit reconstruction method that is exact for noise free continuum data. This situation is similar to exact methods for CT reconstruction, such as filtered back projection in 2D and Katsevich’s method in 3D, in that regularization is applied at an intermediate step. These methods also do not include an explicit forward problem so that the misfit to the data is not calculated and the effect of inconsistent data unpredictable. In EIT, we do not have a complete characterization of the range in 3D, so in contrast to CT it is harder to detect inconsistent data. In EIT and CT, data fitting methods give a reasonable idea of inconsistent data as the residual difference between fitted forward model and measured data will be large. An interesting area for future exploration is the combination of explicit inversion methods such as D-bar with a forward model test consistency, a work we began in this work with the L-curve plots.
